# Parasitoids of Queensland Fruit Fly *Bactrocera tryoni* in Australia and Prospects for Improved Biological Control

**DOI:** 10.3390/insects3041056

**Published:** 2012-10-22

**Authors:** Ashley L. Zamek, Jennifer E. Spinner, Jessica L. Micallef, Geoff M. Gurr, Olivia L. Reynolds

**Affiliations:** 1 Elizabeth Macarthur Agricultural Institute, NSW Department of Primary Industries, Woodbridge Road, Menangle, NSW 2568, Australia; E-Mails: azamek@phau.com.au (A.L.Z.); jess.smart@dpi.nsw.gov.au (J.L.M); 2 EH Graham Centre for Agricultural Innovation, NSW Department of Primary Industries and Charles Sturt University, Locked Bag 588, Wagga Wagga, NSW 2678, Australia; E-Mail: jspinner@csu.edu.au; 3 EH Graham Centre for Agricultural Innovation, NSW Department of Primary Industries and Charles Sturt University, Charles Sturt University, P.O. Box 883, Orange, NSW 2800, Australia; E-Mail: ggurr@csu.edu.au; 4 EH Graham Centre for Agricultural Innovation, NSW Department of Primary Industries and Charles Sturt University, Elizabeth Macarthur Agricultural Institute, Woodbridge Road, Menangle, NSW 2568, Australia

**Keywords:** Braconidae, Tephritidae, *Diachasmimorpha*, *Fopius arisanus*, sterile insect technique, integrated pest management, mass-rearing

## Abstract

This review draws together available information on the biology, methods for study, and culturing of hymenopteran parasitoids of the Queensland fruit fly, *Bactrocera tryoni*, and assesses prospects for improving biological control of this serious pest*. *Augmentative release of the native and naturalised Australian parasitoids, especially the braconid *Diachasmimorpha tryoni*, may result in better management of *B. tryoni *in some parts of Australia. Mass releases are an especially attractive option for areas of inland eastern Australia around the Fruit Fly Exclusion Zone that produces *B. tryoni*-free fruits for export. *Diachasmimorpha tryoni* has been successful in other locations such as Hawaii for the biological control of other fruit fly species. Biological control could contribute to local eradication of isolated outbreaks and more general suppression and/or eradication of the *B. tryoni* population in endemic areas. Combining biological control with the use of sterile insect technique offers scope for synergy because the former is most effective at high pest densities and the latter most economical when the pest becomes scarce. Recommendations are made on methods for culturing and study of four *B. tryoni* parasitoids present in Australia along with research priorities for optimising augmentative biological control of *B. tryoni*.

## 1. Introduction

The Queensland fruit fly, *Bactrocera tryoni* (Froggatt) (Diptera: Tephritidae), is the major fruit fly pest for all of eastern Australia with literature on the species dating back more than 115 years [1]. It is a major economic pest as a consequence of its ability to survive in a wide range of climatic conditions, its polyphagous nature and its destructive damage to most cultivated fruits and fruiting vegetables. The native range of *B. tryoni* is considered to be tropical and subtropical coastal Queensland (Qld) and northern New South Wales (NSW), however, its distribution now extends along much of the eastern seaboard and areas of inland NSW and Victoria [1]. Within NSW, *B. tryoni* is best suited to the climate of the coastal and northern inland areas. It can, however, thrive in less suitable areas such as the south and south-west of the state during years of favourable rainfall, with distribution shrinking back to irrigated areas during dryer years [2]. The majority of *B. tryoni* adults are believed to disperse up to 1 km, although larvae are readily transported in vehicles within infested fruit that pose a threat to many quarantined production areas within suitable climatic zones [3] such as those within NSW. *Bactrocera tryoni* also has the potential to spread internationally because of its tolerance of a wide range of climatic conditions and large host range, as well as its tendency to be dispersed by humans at the larval stage inside infested fruit [3].

The expansion of *B. tryoni *within Australia began as rainforests were cleared and large areas, including inland irrigation zones, were planted with susceptible fruit crops. The largely unrestricted interstate trade of fruits during the late 1890s also contributed to this pest’s spread. Infested fruits quickly become rotten and inedible causing considerable losses in production, often resulting in complete destruction of fruits rather than only cosmetic damage as is caused by many other insect pests [4]. Currently, *B. tryoni *is managed using mainly surveillance (trapping), bait spraying, the sterile insect technique (SIT) and chemical control as well as public education [5,6]. However, with diminishing pesticide options for the control of *B. tryoni*, industries are increasingly looking at other alternatives. The aim of this review is to draw-together available information on the biology, methods for study, and culturing of hymenopteran parasitoids of the Queensland fruit fly, *B. tryoni. *In order to assess prospects for improving biological control of this serious pest in Australia, the international literature on biological control of other fruit fly species is synthesised*.*

## 2. Biological Control Strategies

Natural enemies when applied properly are promising, environmentally-friendly and effective tools for sustainable control of arthropod pests to the extent that biological control of insect pests is one of the most cost effective and environmentally sound methods of pest management [7]. In the past 120 years, more than 200 species of exotic arthropods have been introduced on more than 5,000 occasions into 196 countries for the control of insect pests [8]. Such inoculative or classical biological control offers the advantage that well-chosen agents, compatible with the conditions into which they are released, have the ability to maintain self-perpetuating populations from generation to generation [9], providing good continuity of control. There is always a risk however, that the agent will attack non-target species. Parasitoids are, therefore, often considered a better option than predators, as the former rely on the host for development and are often more host specific [10] so reducing the risk of the agent attacking non-target species. Parasitoids are categorised based on factors such as egg development pattern in the adult female (synovigenic *versus* proovigenic), whether larvae develop within the host (endoparasitoid) or externally (ectoparasitoid), whether the host continues development (koinobiont parasitoid) and feeding after parasitism or is arrested (idiobiont parasitoid), and whether the adult parasitoid feeds upon the host [11]. Many parasitoid wasps (including Braconidae) have the advantage of being self-dispersing giving wide coverage in areas where other techniques such as spraying cannot be readily applied [12]. In addition, parasitoids are in no way dangerous to human health making them an attractive option for fruit fly control in urban areas such as those found in the Risk Reduction Zone (RRZ) in NSW and Victoria [13]. 

Another response to the potential risks of exotic biological control agents is the use of conservation biological control. Conservation biological control aims to maximise the impact of existing natural enemies and has proven effective in many crop/pest systems [14]. There has been little research attention devoted to conservation biological control against fruit flies so the major focus of the present review is in other forms of biological control. A general limitation of many forms of biological control, however, is that the natural enemies will not typically provide adequate pest suppression alone (including braconid parasitoids), thus integration with other pest management tools such as the sterile insect technique (SIT) [9,15] or bait sprays [16] is required. The use of an integrated pest management (IPM) approach is especially important in eradicating local outbreaks of *B. tryoni* in Australia [6,17].

## 3. Historical Use of Biological Control for Fruit Flies in Australia

**Figure 1:**
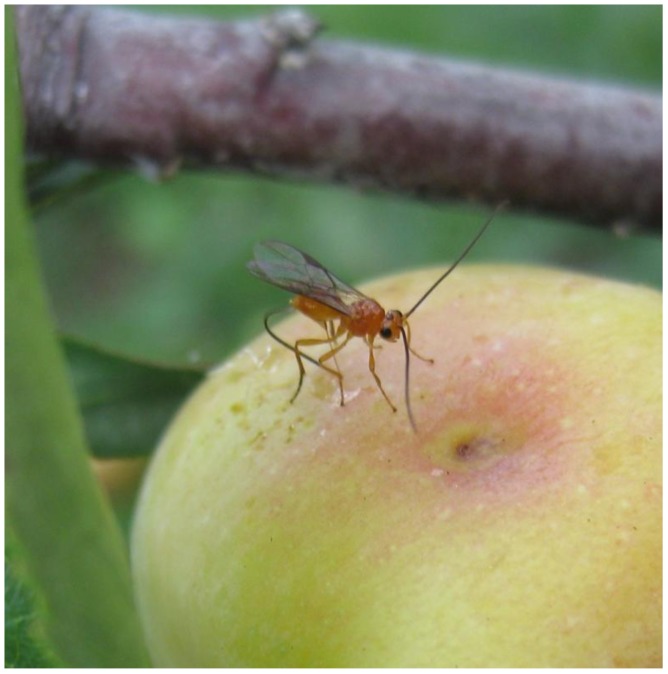
A female *Diachasmimorpha* sp.

Of the eight opiine braconids that occur in Australia and are known to attack *B. tryoni* [18], there are four that are of particular interest for use in augmentative release, not just in Australia but worldwide due largely to their ease of rearing, range of target hosts, climatic/environmental tolerance and levels of parasitism achieved. These four opiine braconids attack a range of tephritid pest species in other locations (Table 1), including several species that are considered a biosecurity risk to Australia. *Fopius arisanus* (Sonan) is an egg-pupal parasitoid [19]; *Diachasmimorpha kraussii* (Fullaway) and *Diachasmimorpha tryoni* (Cameron) target late second to early third instar larvae, while *D. longicaudata* (Ashmead) target third instar larvae [20,21,22] (Figure 1). Of these, only *D. kraussii* and *D. tryoni* are native and have been detected from far north Qld. [18] to southern inland NSW [23]. *Fopius arisanus*, although originally from Malaysia, was introduced from Hawaii and ranges from far north Qld, as far south as Sydney [18]; while *D. longicaudata* also introduced from Hawaii, has been recorded in far north Qld and Lord Howe island [18].

**Table 1:** Host records of the opiine braconids, *Diachasmimorpha *spp. and *Fopius arisanus *parasitising major tephritid pests that occur worldwide. (- indicates no record).

Major Tephritid Pests	Major Opiine Braconids			
	*D. longicaudata*	*D. kraussii*	*D. tryoni*	*F. arisanus*
*Anastrepha ludens *(Loew) (Mexican fruit fly)	[24]	-	[24]	[24,25]
*Anastrepha suspense *(Loew) (Caribbean fruit fly)	[26]	-	-	[25]
*Bactrocera cacuminata *(Hering) (Wild tobacco fly)	-	[18,22]	-	[18,25]
*Bactrocera cucurbitae *(Coquillett) (Melon fruit fly)	[18]	[18]	-	[25,27]
*Bactrocera dorsalis *(Hendel) (Oriental fruit fly)	[18,28]	[19,22]	[18]	[18,19,28]
*Bactrocera latifrons *(Hendel) (Malaysian fruit fly)	[18]	[29,30]	-	[18]
*Bactrocera oleae *(Gmelin) (Olive fruit fly)	[31]	[31]	-	[25,32]
*Bactrocera papaya *Drew & Hancock (Papaya fruit fly)	-	-	-	[25]
*Bactrocera tryoni *(Froggatt) (Queensland fruit fly)	[18]	[18]	[18]	[18,25]
*Ceratitis* *capitata *(Wiedemann) (Mediterranean fruit fly)	[18]	[30,33]	[18,19]	[18,33]

The first effort to introduce braconids into Australia was in 1902 for the control of the Mediterranean fruit fly (*Ceratitis capitata* Wiedemann) in Western Australia after unsuccessful searches for native natural enemies of this pest [33]. Between 1932 and 1938, the NSW Department of Primary Industries (DPI) attempted biological control of *B. tryoni* with introductions of several thousand *Tetrastichus giffardianus *Silv., and small numbers of *Opius humilis *Silv. and *O*. *fullawayi *Silv. [34]. Later, large numbers (over 205,000 in NSW alone) of *Melitobia* (*Syntomosphyrum*) *indicum *Silv. [34] were released, however, all of these biological control attempts failed with none of the released species establishing [34]. There is no published literature documenting augmentative release of mass-reared parasitoids, whether native or exotic, against *B. tryoni* in Australia.

## 4. International Use of Biological Control for Fruit Flies

Utilising parasitic wasps for the control of fruit flies dates back to the early 1900s [18]. Initial attempts were centred on classical biological control whereby parasitic species associated with the target pest in its place of origin were inoculated into new geographical locations. For example, *D. longicaudata*, originally from south-east Asia [35], was successfully introduced into Hawaii [36] and subsequently into Australia from Hawaii in 1956–1957 [37]. This species is now widely established in the east of Australia, including Lord Howe Island [18], as well as in Florida (USA), the south of Central America and throughout South America [38,39]. *Fopius arisanus*, also native to south-east Asia [35], was established in Hawaii and later Australia (1956–1957) [28]. Its introduction into other parts of the world including Israel, Mexico, and South America, however, was less successful, except in Costa Rica where it has achieved patchy establishment [33,39,40]. 

Other examples of parasitoid species used in classical biological control include the native Australian species *D. kraussii* and *D. tryoni* [18]. *Diachasmimorpha kraussii* has been successfully introduced to Israel and Hawaii [33,41] and while approximately 15 *D. tryoni *were initially released in Maui, Hawaii, around 4.1 million were later augmentatively released [42] for control of *C. capitata* [24,43,44]. Shortly after its introduction, *D. tryoni* became the most abundant parasitoid in Hawaii [45], comprising up to 33% of total parasitoids in Kula, Maui [44]. Mass releases of *D. tryoni* in Mazapa de Madero Canyon in Mexico between 1987 and 1989 have also been successful, substantially reducing infestations in mangoes and oranges and greatly decreasing populations of *Anastrepha ludens *(Loew) and *Anastrepha oblique * (Macquart) [39].

In recent decades, increasing interest in the active integration of parasitoids into integrated pest management programs has replaced classical biological control. This is likely to reflect not only an increased awareness of the risk of non-target impacts but also significant advancements in rearing techniques and artificial diets for rearing hosts for augmentative biological control programs [46,47]. These advances have allowed parasitoid wasps to be used as biological control agents against various fruit fly pests in augmentative release programs [48,49,50]. Augmentative biological control involves the supplemental release of parasitoids and relies on the mass-production of large numbers of parasitoids in a laboratory. Relatively few natural enemies may be released at a critical time of the season (inoculative release) sometimes with the expectation that reproductive populations will establish or literally millions may be released (inundative release). In addition, the crop/host plant system may be modified to support or augment the parasitoids, often known as habitat manipulation or cultural management. Parasitoids have been used successfully in augmentative release programs in Hawaii, Mexico, Guatemala and Israel [33,39,51]. The major species utilised in augmentative research and control programs worldwide are *D. kraussii*, *D. longicaudata*, *D. tryoni *and *F. arisanus* with the expectation that the released parasitoids will suppress the targeted pest. A particularly successful example of augmentative biological control of fruit flies occurred in Hawaii where parasitoids dramatically reduced fruit fly (mainly *Bactrocera dorsalis *(Hendel) and *C. capitata*) densities within one year [52]. Trials of *C. capitata* control in Hawaii using the native Australian parasitoid, *D. tryoni* involved releasing 4.2 million parasitoids, averaging 265,000 per week, resulting in significantly lower *C. capitata* per fruit compared to an untreated control area [50]. 

Introducing biocontrol agent species to new habitats, especially in such large numbers, may raise host specificity risks [9]. For example, imported parasitoids have been reported to form new host associations [53], sometimes with other introduced species [54]. Since the introduction of *D. tryoni *to Hawaii for the control of *C. capitata*, this parasitoid has formed host associations with two gall‑forming tephritids, the lantana gall fly, *Eutreta xanthochaeta *Aldrich, and the Pamakani gall fly, *Procecidocharea utilis *Stone, introduced to Hawaii for the control of weeds [54]. These new host associations of *D. tryoni* are thought to possibly be due to the introduction of *F. arisanus*, a competitor of *D. tryoni. Diachasmimorpha kraussii *was also introduced to Hawaii for the control of *Bactrocera latifrons *(Hendel), the solanum fruit fly. Tests of possible host associations with native and introduced tephritid fruit flies in Hawaii found that gravid *D. kraussii *females would oviposit into all host larvae presented [29]. These studies were performed under confined laboratory conditions in choice and no‑choice scenarios, and while it is not known whether such host associations occur in the field, the aforementioned findings illustrate the potential implications (e.g., unfavourable new host associations) of introducing a species into a new habitat and the desirability of using native parasitoids over exotics. 

## 5. Mass Production of Parasitoids

A limiting factor of mass-rearing most species of parasitoids (and all that attack fruit flies) is that it requires the use of live hosts, which in turn, increases production costs [55]. The quality (weight and size) of the host must be appropriately monitored as parasitoid cultures from low quality hosts tend to have a male biased sex ratio [56]. Hymenopteran parasitoids are haplodiploid with infertile eggs producing males and fertilised eggs producing females [57]. Rearing facilities aim to optimise production of female parasitoids as females are the sex responsible for exerting biological control of the target species.

The size of host larvae is also a crucial factor for parasitoid emergence. High quality hosts that allow production of comparatively large fruit fly larvae increases parasitoid emergence [56]. The age and condition of the larvae used during the rearing process is an important factor influencing both percentage emergence and sex ratio of mass reared parasitoids [58,59]. Measurements used to monitor the quality of mass reared parasitoids include: host weight, adult emergence, survival, fecundity, flight and searching behaviour [60]. Such monitoring is important because the performance of mass reared insects produced for augmentative biological control can decrease over generations due to their adaptation to laboratory conditions [61]. These changes may decrease their performance in the field and reduce the success of a parasitoid in a particular biological control program [61]. Therefore, various aspects of parasitoid biology need to be considered in order to optimise the rearing of parasitoids in large numbers. 

### 5.1. Mating Behaviour

Many insect species have adult female parasitoids that respond to air- and substrate-borne male courtship signals [62] that are pivotal to successful mating. Males usually emerge a few days before females so are ready to mate immediately when females emerge, although the optimum mating activity occurs when females are 3–7 days old [46]. Five species of male parasitoids including *D. tryoni* and *D. longicaudata*, exhibit pulses produced by male wing fanning that are repeated numerous times during courtship [61]. A constraint in mass rearing is that the artificial rearing environment can affect the transmission and detection of these important courtship vibrations and thus impact rearing efficacy. Mass production can also lead to selection for mating traits that are adaptive in the rearing facility but that can adversely impact subsequent success of the female wasp when released in the field [61]. 

### 5.2. Substrate Cues and Oviposition

Successful host location is a major problem for gravid parasitoids. Consequently, the exploitation of cues associated with the presence of hosts is very important in parasitoid foraging [63] and determines their success in both mass rearing and the wild. Both host larvae and their associated substrate (fruit), produce cues that trigger ovipositor-probing behaviour in gravid parasitoids [62]. The cues from host larvae are mainly vibrations and/or sound created by larvae feeding and crawling inside the fruit. The cues from the host substrate are probably contact short-range volatile chemicals originating from the fermentation process caused by larval damage and/or the excrement of feeding larvae. Frugivorous tephritid larvae frequently cause bacteria-related decay of fruit, which subsequently emit chemical cues that may be used by parasitoids to search for hosts within the fruit. Chemical cues from the fermented host substrate are just as important as host vibration cues in the host searching process [62]. These chemical and/or physical cues associated with the infested fruit or produced by the feeding larvae are critical stimuli which allow female wasps to find and recognise infested fruit and available hosts [43]. If these important host cues are absent in mass rearing, or not recognised as a result of the rearing environment, the parasitoids will not commence oviposition behaviour [46]. 

Local arrestment of a foraging parasitoid occurs after a host-infested fruit has been located via chemical cues [62]. The parasitoid female then uses her antennae to perceive locally high concentrations of the chemical cues produced by the host infestation. Parasitoids may respond to the chemical cues by increasing their rate of turning or reducing their walking speed. Vibrotaxis involves the parasitoid standing stationary on the fruit surface and using its legs to perceive vibrations caused by a feeding or moving host larva. In response to detecting vibrations, the parasitoid will probe that particular area with the ovipositor. Ovipositor probing is an essential element of host-searching behaviour [44] and commences only after successful perception of other, preliminary cues. Accordingly, ovipositor probing is used as an indicator of the level of acceptance of a specific host‑substrate [43]. 

### 5.3. Emergence and Pre-Release Interventions

Host pupal weight is positively correlated with emergence percentage (e.g., *D. tryoni* [46]), while high larval weights produce larger adult parasitoids [64]. Larval weights greater than 4 mg are considered optimal in mass-rearing to ensure maximum emergence of fruit fly parasitoids [46]. A problem that can prevent parasitoid development and emergence is encapsulation. Encapsulation is the process whereby haemocytes form a multi-layered envelope around the invading organism [65]. Egg encapsulation is a typical immune response by host insects in response to attack by parasitoids and has been recorded in numerous host species [65]. Parasitoid eggs laid in hosts, other than the natural hosts are usually killed by encapsulation [44]. Advances in rearing techniques include the use of irradiated larvae which improves parasitism rates by compromising the hosts’ immune system [66], reducing the likelihood that immature parasitoids will be encapsulated. Irradiation of hosts also offers the advantage that only parasitised hosts will emerge and flies that are not parasitised fail to develop [67,68]. The effect of a sublethal does of radiation is usually not apparent until the next moult [69]. Radiation affects actively growing cells and large molecules of DNA [70] and as dose increases, damage caused to the cells and DNA makes it impossible for an adult fly to develop. This is important because rates of parasitism are rarely 100% and the escape or release of unirradiated or non-sterile adult hosts from a parasitoid rearing facility could contribute to pest impact. Purcell [46] recommends a ratio of host larvae to female parasitoids of at least 15:1 in order to avoid hosts being attacked by multiple ovipositing wasps. Such super-parasitism can cause parasitoid mortality, low emergence rates and poor host quality in mass rearing [13], however, recent studies have demonstrated that moderate levels of superparasitism by *D. longicaudata* in the Mexican fruit fly, *A. ludens*, may result in a female-biased sex ratio with few negative effects on the offspring [71].

Mass-release of parasitoids involves transporting parasitoids to the intended area of release, a process that may take several days, during which parasitoids may suffer a decline in viability. In order to avoid this, parasitoids may be chilled (3.5–4.5 °C). Larios *et al.* [72] reported that there was little or no adverse effect of chilling on parasitoid longevity, production of daughters or offspring sex ratio. Methods of release for *D. tryoni* now include chilling followed by aerial release, similar to the method used to dispense chilled sterile fruit flies for SIT [73]. Chilling has no discernable effect on the short‑term mortality of *D. tryoni* or on its ability to take flight immediately after aerial release [72].

The behaviour (e.g., foraging) of a parasitoid is directly linked to their nutritional state [74,75]. In addition, food is an essential component in maximising the reproductive success of adult female parasitoids [76]. The pre-release environment provides opportunities for interventions, including pre‑release feeding to maximise longevity and fecundity [74,77], and the exploitation of parasitoid learning ability to maximise the chance of locating and parasitising a host [78,79,80]. Pre-release feeding of parasitoids offers scope to maximise their performance following release, however, few studies have investigated the effect of different pre-release food types on parasitoid longevity in the laboratory or in the field. One such experiment used honey-water (50% solution by volume) as the food source to test the mating status and oviposition of *D. kraussii* and *D. longicaudata* and determine the effect on parasitoid longevity [31]. Zamek *et al. * [81] provided 10% solutions of several diets to *D. tryoni*. Adult females fed white sugar had the longest lifespan whilst honey and golden syrup (56% invert syrup (glucose and fructose), 44% sucrose) resulted in similar survivorship curves. This suggests that there is a need to compare white sugar (and other food types) at varying concentrations with the current common practice in many mass-rearing facilities worldwide of providing pure honey to fruit fly parasitoids. Finding suitable hosts in which to lay their offspring is a difficult task for female parasitoid wasps. In order to overcome this location problem, female wasps use stimuli that are derived from their hosts or that indicate suitable hosts [82,83]. The use of indirect, chemical information is an important solution for parasitoid wasps to be able to detect their hosts. Parasitoids of dipteran hosts use odours of decaying substrates like fruits or carcasses to find host fly larvae. In parasitoid wasps, learning of odour cues has been the focus of most studies (e.g., [83,84]), however parasitoids are able to learn other cues such as colours, patterns, shapes and spatial information (e.g., [82,85,86]). Parasitoids may also use learned information about the availability of suitable hosts and use that information during successive visits to other areas. For parasitoids of dipteran larvae, previous experience with unparasitized hosts reduces the acceptance of ensuing hosts that already have been parasitized [87]. Parasitoids can change their innate preferences for odour cues that guide them to areas with hosts after an oviposition experience, permitting female parasitoids to find hosts sooner; possibly increasing their lifetime foraging success [88]. Although learning in parasitoids is not a new area of study (for review, see [78]), the exploitation of the learning abilities to enhance parasitoid searching success in a release program is a novel aspect requiring further investigation.

### 5.4. Release Rates and Monitoring

Release rate is also pivotal to successful augmentative release programs. In a modelling study, on no specific parasitoid species, Knipling [89] predicted a release ratio of a larval parasitoid to pest at 3.3:1 would result in 96% parasitism, however, in practice, Sivinski [90] preferred a ratio of parasitoid (*D. longicaudata*) to pest (*A. suspensa*) of 10:1. Knipling [89] draws attention to the notion that different parasitoid/pest complexes will require different ratios. Control programs, therefore, need to be efficient and developed with adequate monitoring tools. Traditional monitoring methods for parasitoids rely on rearing and dissection of host material for identification and quantification of parasitism [33,50,91]. These tasks can be tedious, as *B. tryoni* does not emerge until fourteen days after pupation and parasitoids at least a further two days. Furthermore, related parasitoid species often have few or no morphologically distinguishable features in the immature stage [91], making it impossible for identification after dissection. In systems where quick identification and quantification of parasitism levels are required, such as in augmentative releases programs, molecular diagnostic tools would be of great use [92]. With rapid advancements of molecular genetics, a new wealth of techniques have been developed that are relevant [92,93] and applicable for augmentative release program monitoring. Data, such as current parasitism levels, could be quickly processed, and a decision made to release further parasitoids if required. Molecular techniques have been used to monitor the incidence of parasitism in other species [93], however, they have not been applied to the parasitoids of *B. tryoni* nor has molecular identification been implemented as a monitoring tool for *B. tryoni* parasitism in field collected samples [92]. 

A final problem associated with mass-production and augmentative release of biological control agents is that the large numbers of released insects can modify the genetic structure of the wild population by mating with wild individuals. This “reverse bottleneck” is an instance in which the genetic structure of a population is diluted in the presence of a more numerous domesticated population of differing genetic structure [42]. This effect can be overcome by regularly infusing laboratory cultures with fresh ‘wild’ specimens to ensure the genetic diversity of the original wild population is maintained.

## 6. Prospects for Parasitoid-Based Biological Control of Fruit Flies in Australia

Despite the success of parasitoids as agents in international augmentative tephritid biological control programs, parasitoids have not yet been used for augmentative release in Australia against *B. tryoni* or *C. capitata*, the two key economic fruit fly pests in Australia. Mass-rearing facilities for *B. tryoni* and *C. capitata* are located in NSW and Western Australia, respectively. These facilities currently produce flies for the sterile insect technique (SIT), and could be a source or expand production to supply host material for parasitoid rearing. As 70% of the cost of parasitoid rearing is associated with the production of host material [94] the existence of host rearing infrastructure and expertise in Australia is a benefit to the economics of parasitoid rearing. 

Information on the biology of parasitoids is of great value in developing and improving mass‑rearing techniques and augmentative release programs [95]. Accordingly, to assess the potential utilisation of available parasitoid species for biological control of fruit flies in Australia, the following sections review key aspects of parasitoid biology, together with available information on the methods that are used for the study of fruit flies and their parasitoids, focusing particularly on the four major opiine braconids, *D. longicaudata*, *D. kraussi*, *D. tryoni*, and *F. arisanus *that are currently being considered for augmentative biological control.

### 6.1. Suitability of Parasitoid to Release into the Environment

#### 6.1.1. Climate Matching

Information on the thermal biology of parasitoids is important in assessing their utility in augmentative biological control as this allows their effective range to be determined by using models such as CLIMEX. A complementary approach for assessing the zones in which a given parasitoid may have potential as an augmentative biological control agent is to study their spatial and seasonal abundance. This can indicate currently unoccupied areas into which the parasitoid could be released and is of particular relevance to Australia, where *B. tryoni* exists in some regions as a meta-population. This is a result of the large size of the country, highly variable climatic zones, presence of areas of irrigated horticulture and towns in otherwise uninhabitable arid vegetation, combined with high levels of human movement for commerce and leisure.

The distribution of *D. longicaudata* is temperature delimited to areas with mean temperatures above 10.5 °C and below 30 °C, despite the presence of its host in the region at these temperatures. The longevity of adult *D. longicaudata* is reduced to 3 days at constant temperatures above 30 °C [31]. At moderate temperatures (15 °C to 25 °C), longevity decreases with increasing temperature (from 19 days down to 7 days, respectively). Caution is, however, required in interpreting the results from such laboratory studies in which constant temperatures are used. Diurnal fluctuations in the field are likely to allow parasitoids respite and allow foraging for hosts [31]. This is important because summer temperatures in some *B. tryoni*-infested zones of Australia can exceed 30 °C. An additional point of caution regarding the longevities cited in the aforementioned study is that humidity was not regulated in the experiments. Future work should aim to match humidity levels to those likely to be encountered in the field as this can strongly affect longevity. For example, *D. kraussii* survived up to 30 days at 25 °C and 60% relative humidity (RH) [22], surviving significantly longer than when humidity was not controlled [31].

The longevity of *D. kraussii* also decreases with increasing temperatures over the range likely to be encountered in the field in Australia (e.g., from 36 days to 11 days at 15 °C to 25 °C) [31]. While both adult *D. longicaudata* and *D. kraussii* can survive for only 3 days at constant temperatures above 30 °C, *D. kraussii *is more likely to be able to survive high temperatures than *D. longicaudata *by resting in the heat of the day and foraging at cooler times [31]. Information is sparse for *D. tryoni* and *F. arisanus *but it has been reported for the latter that it does not survive below 15.5 °C [96], although maximum threshold temperatures have not been studied. The impact of high temperatures on all of these species could be ameliorated to some extent, however, by allowing adult parasitoids to feed and mate prior to releases being made in summer months. Potentially, adults should also be held for several days under favourable storage conditions to allow egg maturation so that adult females are able to parasitise available hosts immediately upon release. 

#### 6.1.2. Field Sampling

The study of parasitoids and their potential as biological control agents is underpinned by field surveys in which the parasitoid fauna is determined. Because of its fundamental importance, the literature on sampling methods is detailed. Fruit harbouring hosts, and potentially parasitoids, can be collected from research orchards [21], backyards [28,97,98], or bushland [97,98]. Fruit has rarely been sampled from commercial orchards because pesticides used in these settings, along with the practice of collection and destruction of fallen fruit, are likely to lead to low parasitoid densities [99]. Parasitoids can be collected by direct capture of adults in the field, utilising some form of aspirator [21] or rearing them from parasitised hosts within infested fruits. The latter collection technique is often preferred as this will also indicate the identity of hosts [21,98,100,101].

Due to the polyphagous nature of most tephritids, both native and exotic fruit trees need to be surveyed [98,99]. As host plant species fruit at different times of the year and for different lengths of time, sampling must reflect such temporal trends. It is important, therefore, to build up a database of trees in the sampling area and revisit these sampling sites throughout the fruiting season. Sampling needs to include fallen fruit as well as fruit on the trees as different host insects preferentially target these resources [73,97,98]. Lopez *et al.* [55] advocate climbing trees or the use of a ladder, with fruit collected in a bucket attached to a pole. In a research orchard, this may be practical; however, it may be cumbersome when collecting in multiple locations throughout the day. Fruit ready to abscise will give the best indication of natural parasitism levels as the pest larvae in the fruit have been exposed to parasitoids for the maximum possible period of time [55,66]. In order to determine if fruit is ready to drop, tree branches can be gently shaken [55,66]. Picking fruit that is not completely ripe can lead to an underestimation of parasitism rates due to the decrease in the period where larvae are susceptible to attack, with some larvae having never been vulnerable to attack (dependent upon oviposition preferences of parasitoid species present) [47,98]. This also allows a shorter rearing time as the larvae are more likely to be near pupation [55]. In order to alleviate this issue, Wong *et al*. [50] dissected fruit at collection, removing mature larvae for rearing. In a different approach, Sivinski [66] considered only larvae which exited fruit within three days of sampling. 

Collections have been made at weekly [98] to monthly intervals [102] for periods of seven months [23], twelve months [99] to two years [98]. Sample size differed with quantity and fruit varieties available [97,103] or time spent sampling [102]. Sample size ranged from five per tree per week [98] to 2–100 pieces of fruit of each type of fruit available [28]. The concurrent collection of site and management data is important for the full interpretation of trends. For example, any pesticide applications need to be noted [55].

In tropical regions especially, native fruit trees may act as alternate hosts for pest fruit flies and therefore require sampling for parasitoids [98]. Longer fruiting seasons in these regions are also likely to lead to higher numbers of parasitoids, as well as pests, and would be good locations for collections when the aim is to establish or supplement parasitoid colonies. In more temperate regions, such alternative host plants may be less readily available. There may however, be non-pest species of fruit flies that act as hosts for parasitoids when the major economic pest is not available. 

##### 6.1.2.1. Sentinel Fruits

The use of sentinel fruits, fruit items infested with host larvae and placed out in a sampling pattern and collected later for study, can be particularly useful for determining whether a parasitoid species prefers to forage within the canopy or on fallen fruit [55]. There is conflicting evidence in the literature as to whether or not sentinel fruits maximise the general efficiency of collection of parasitic wasps. It was suggested by Jessup and Walsh [21] that the use of sentinel fruits optimises the collection of wild parasitic wasps due to the extended amount of time the fruit remains in the field. However, Hernandez‑Ortiz *et al.* [99] found that fruit picked directly at the site had parasitism rates more than twice as high as sentinel fruits (68.5% *versus* 31.5%). 

Sentinel fruits should ideally be placed in the field within a layered container system, consisting of one vessel with holes above another containing a pupation medium. Protection from rain is provided by a final container or sheet of fiberglass or similar material. A gap of 50–100 cm will allow parasitoids to detect the fruit fly infested fruit and enter [21,55]. Adhesives such as Tanglefoot (Grand Rapids, MI, USA) will protect the sentinel fruits from ants [55]. The use of a cover is important to prevent sunburn of sentinel fruits [21]. Generally, the use of sentinel fruits is best restricted to sites with controlled access, where human disturbance is unlikely. 

##### 6.1.2.2. Sample Processing

Samples can be returned to the laboratory in labeled paper bags [98], labeled plastic bags [104] or directly into the vessels in which wasps will be reared-out (see below). Where possible, fruit should be transported to the laboratory on a daily basis [55] in order to reduce the risk of spoilage. To determine the identity of host fruit flies, fruits should be placed in individual containers [55] until the emergence of pests or parasitoids [55,73]. Moistened vermiculite [21,102], sand [73,100] or soil [99] can be used as a pupation medium, usually with a depth of 1 cm [55,73,98]. Ventilation of containers can be achieved by cutting a hole in the container lid and covering with mesh [21,55,73]. This also prevents flies and wasps escaping and *Drosophila* spp. from entering and contaminating the culture [105]. Where only one species of fruit fly is expected or where host records are not of concern, fruit can be placed in bulk lots consisting of a single fruit type from each location [98,100]. Whether rearing out in individual or bulk containers, it is preferable to elevate fruit above the pupation medium [21,100]. This can be achieved by using sieves with holes large enough to allow larvae to pass through after exiting the fruit [100], wire mesh [21], or suspending the fruit with netting [104]. 

##### 6.1.2.3. Sample Maintenance

Recommendations on sample maintenance vary from daily [102], to once a week [21]. Fruit should be checked for mould and moistness of media regularly, but may only need attention at longer intervals [73,98]. Several researchers, including Sivinski *et al.* [73], Aguiar-Menezes *et al.* [97] and Mkize *et al*. [100], advocate sifting media every second day and counting or moving pupae to fresh vermiculite in a separate rearing container. This practice, however, is very time consuming for large scale survey work. At 26 °C, all host larvae will leave the fruit within two weeks [106], however, if the fruit is completely covered with mould it should be dissected earlier to determine if all larvae have emerged [55,73]. Earlier dissection will prevent fruit rotting fungi (e.g., *Penicilium* spp.) progressing to attack the pupae. If larvae are not found in the fruit, the fruit sample may be discarded. If immature larvae are found, the fruit, or the least non-rotten portion, should be returned to the container to allow the larvae to complete their lifecycle [105]. Artificial diets are useful for rearing larvae recovered from decomposing fruit, but if only a few fruits yield larvae, it may not be economical. Emergence of flies and wasps should be assessed every three days [55] for approximately four weeks [21,26]. Many parasitoid species enter diapauses so uneclosed pupae should either be retained for up to twelve months [73] or dissected to identify the developing imago. Wing venation, however, is an important distinguishing feature between the braconid genera *Fopius* and *Diachasmimorpha* [18]. Therefore, it is preferable to allow natural eclosion of adults so that this identification feature is well developed. Dead pupae can be reconstituted by soaking in water for 48–96 hours. Dissection of the re-hydrated pupae enables detection of at least basic features that will discriminate flies from wasps [29]. Alternatively, molecular analyses for identification can be used in this situation [92].

### 6.2. Host Range and the Risk of Non-Target Impact

*Fopius arisanus*, *D. kraussii*, *D. longicaudata* and *D. tryoni* all attack a wide range of tephritid fruit flies, including species native to and exotic to Australia, not all of which are pests. Potentially a wide host range facilitates biological control by allowing a parasitoid to persist and reproduce in an area during times of local scarcity of the target (pest) by exploiting other (non-pest) host species. Offsetting this, however, is the fact that risk to a non-target host can make regulatory authorities reluctant to allow biological control programs. This is certainly the case when a classical biological control introduction is being considered but may also apply to augmentative biological control that will increase local parasitoid abundance and increase the magnitude of risk.

#### 6.2.1. Non-Target Impacts

Tephritidae are grouped into several sub-families and include fruit feeders (commonly referred to as fruit flies), gall-formers and flower-feeders [54]. Tephritid flies are parasitised by wasps in the subfamilies Opiinae (Fam. Braconidae), Dirhininae (Fam. Chalcidae), Euderinae, Tetrastichinae and Entedoninae (Fam. Eulophidae) [107]. It is commonly assumed that the opiine braconids coevolved with their frugivorous tephritid hosts [35], however, there are reports of opiine braconids also parasitizing gall-formers (Table 2) and flower-feeding tephritid flies worldwide (Table 3). 

**Table 2:** Non-target impacts of opiine braconids, *Diachasmimorpha*, *Fopius*, *Psyttalia*, and *Tetrastichus* spp., on gall-forming tephritids. (- indicates no record).

Host	Parasitoid	Oviposition recorded in gall	Oviposition into larvae in artificial diet	Parasitoid able to complete lifecycle	Threat	Reference
*Phaeogramma lortnocoibon*	*D. longicaudata*	×	×	-	Nil	[52,108]
*Eutreta xanthochaeta*	*D. longicaudata*	✓	✓	-	Very low	[109]
*Procecidochares alani*	*D. longicaudata*	×	✓	×	Low	[52,108]
*Procecidochares utilis*	*D. kraussii*	✓	-	×	Low	[29]
*Eutreta xanthochaeta*	*D. kraussii*	✓	✓	✓	Moderate	[29]
*Phaeogramma lortnocoibon*	*D. tryoni*	×	×	-	Nil	[52,108]
*Procecidochares alani*	*D. tryoni*	×	✓	×	Low	[52,108]
*Eutreta xanthochaeta*	*D. tryoni*	✓	✓	-	Moderate	[108,110]
*Procecidochares alani*	*F. ceratitivorus*	×	×	×	Nil	[111]
*Phaeogramma lortnocoibon*	*P. fletcheri*	×	×	-	Nil	[52,108]
*Procecidochares alani*	*P. fletcheri*	×	×	-	Nil	[52,109]
*Eutreta xanthochaeta*	*P. fletcheri*	✓	×	-	Very low	[112]
*Procecidochares alani*	*T. giffardianus*	✓	✓	✓	Moderate	[52]

**Table 3:** Non-target impacts of opiine braconids, *Diachasmimorpha*, *Fopius*, and *Psyttalia* spp., on flower-feeding tephritids. (- indicates no record).

Host	Parasitoid	Oviposition recorded in flowerhead	Oviposition into larvae in artificial diet	Parasitoid able to complete lifecycle	Threat	Reference
*Trupanea dubautiae*	*D. longicaudata*	×	✓	×	Very low	[113]
*Ensina sonchi*	*D. kraussii*	✓	-	×	Very low	[29]
*Trupanea dubautiae*	*D. kraussii*	✓	-	×	Low	[29]
*Trupanea dubautiae*	*D. * *tryoni*	✓	-	×	Very low	[54]
*Ensina sonchi*	*D.* * tryoni*	✓	-	✓	Low	[54]
*Trupanea dubautiae*	*F. arisanus*	✓	-	×	Very low	[7]
*Trupanea dubautiae*	*F. ceratitivorus*	✓	-	×	Very low	[7,111]
*Trupanea dubautiae*	*P. fletcheri*	×	✓	×	Very low	[113]
*Trupanea dubautiae*	*F. caudatus*	✓	-	×	Very low	[7]

Most research on the non-target effects of opiine parasitoids has been conducted in Hawaii, where there are native and introduced gall-formers, the latter introduced for weed biocontrol [54] and flower feeders which are important pollinators of rare and endemic plants [7]. Four gall-formers have been introduced into Hawaii from Mexico for weed biological control: *E. xanthochaeta *Aldrich, for the control of the woody weed lantana (*Lantana camara *L.), pamakani gall fly, *Procecidochares alani *Stekyskal, for the control of pamakani weeds, and *Ageratina riparia *(Regel) and *P. utilis *for the control of crofton weed, *Ageratina adenophorum * Spreng. The bidens gall fly, *Phaeogramma lortnocoibon *Asquith, is endemic to Kauai Island, Hawaii and is associated solely with the plant, *Bidens cosmoides * (A. Gray) Sherff [52,54,109,112]. There have been a number of laboratory studies into non-target impacts of fruit fly parasitoids with such gall-formers (Table 2). It is suggested that some *Diachasmimorpha* spp. parasitise *E. xanthochaeta *galls due to the favourable ratio of ovipositor length to gall wall thickness [108]. However, in choice tests, *D. kraussii* and *D. tryoni* preferred frugivorous flies in fruit or artificial diet over gall flies [29,110]. When tested against their natal host, *D. tryoni* preferred to oviposit into coffee berries rather than lantana stem galls [110], although wasps emerging from *E. xanthochaeta* probed their natal host more frequently than those emerging from *C. capitata* [114]. Field studies have recovered *D. tryoni*, *Eurytoma tephritidis* Fullaway (Eurytomidae), and *Bracon terryi* (Bridwell) (Braconidae: Braconinae) from mature galls [45]. Field releases of *D. longicaudata *in lantana patches resulted in 0.8% parasitism [109]. These results are supported by field surveys by Duan *et al.* [112] on the parasitoid complex attacking *E. xanthochaeta*, showing *D. longicaudata* to be the least abundant parasitoid. These results and previous data confirm *D. tryoni* as the most abundant parasitoid of *E. xanthochaeta*, comprising over 85% of the parasitoid complex [45,50,115]. *Eurytoma tephritidis*, and *B. terryi* are also major parasitoids of *E. xanthochaeta *galls in Hawaii [50]. *Psyttalia fletcheri *(Silvestri),introduced from India for the control of melon fly, *Bactrocera cucurbitae* Coquillett, has never been recorded parasitising gall-forming tephritids in Hawaii [115]. *Eutreta xanthochaeta* galls harvested near *D. tryoni* releases and in non‑release areas showed no significant increase in parasitism of galls when 100,000 *D. tryoni* adults were released per hectare per week [50]. *Diachasmimorpha tryoni* parasitised significantly more *E. xanthochaeta* as the elevation increased and land use moved from agriculture to native forest [45]. Overall, *D. tryoni* was more abundant in the highland habitat areas [36,116]. In this cooler, more humid area, lantana was much more abundant (and therefore the number of galls present were higher), providing increased off-target opportunities, however, there was also an abundance of strawberry guava trees, *Psidium littorale *var. *cattleianum* (Sabine) which harboured *C. capitata* and Oriental fruit fly, *B. dorsalis*. Regardless of larval host, gravid females strongly favoured fruit hosts to lantana stem galls. The finding that *D. tryoni* incidence was correlated with site but not with gall density [45], supports the theory of climate preference and presence of the usual host, rather than preference for gall flies. Duan *et al.* [45] suggest that the parasitoids are attracted to the habitats of the preferred host, where less preferred hosts may also be present. 

Laboratory studies also show scope for non-target impacts of fruit fly parasitoids on flower-feeders (Table 3). Generally, flower-feeding tephritids are at less risk of attack by opine parasitoids than are gall-forming tephritids and it has been suggested that galls are more attractive to fruit fly parasitoids as they are more similar in shape and size to fruit than flowerheads [113]. The number of visits to, and probing of flowerheads, was low and not significantly different in the presence or absence of normal fruit fly hosts [7,54]. The exception is *E. sonchi *and *D. tryoni*, with the presence of *C. capitata *significantly decreasing off-target interactions [54]. Whilst these braconids are not likely to affect *T. dubautiae *and *E. sonchi* in the field, the chalcids *Habrocytus elevatus *(Pteromalidae) and *Euderus metallicus *(Eulophidae) have been observed to attack *Trupanea *spp. in the wild [113].

Other than extrapolation from the international studies reviewed above, there is little information available on the risks of non-target effects in Australia. Flower-feeding tephritid genera in Australia comprise *Dioxyna*, *Tephritis* and *Trupanea *spp., which feed on Asteraceaous plants, and *Oedaspoides*, which feed on Goodeniaceae plants [107]. There is no information, however, on attack, if any, by parasitoids. In the case of gall-forming tephritidae, there is anecdotal evidence of the parasitoids *D. tryoni* and *D. longicaudata* (both present in Australia) attacking *P. utilis *in Hawaii [108]. The latter tephritid was introduced to NSW and QLD for the biological control of crofton weed [107]. Although there do not appear to be any records of attack in Australia, the risk of attack could be elevated if augmentative releases were made and is indicative of the more general need for caution in the use of these parasitoids in biological control. The vulnerability of a non-target host to a parasitoid depends upon the attractiveness of a host for oviposition and its physiological suitability for the completion of the parasitoids’ lifecycle [109]. These biological factors, which also have a bearing on the more general suitability of the parasitoid for mass rearing and use in augmentative biological control, are examined for species of relevance to Australia in the following sections.

### 6.3. Parasitoid Biology

#### 6.3.1. Fecundity

*Diachasmimorpha longicaudata* compares favourably with other parasitoids for reproductive output, regardless of host. When reared on *A. ludens*, *B. dorsalis* and *B. oleae*, *D. longicaudata* produced 187, 93 and 24 offspring per female, respectively. When reared on *A. ludens*, *F. arisanus* produced less than half of this number of offspring (71 offspring), whilst *D. tryoni* produced just under half as many offspring as *F. arisanus* (39 offspring). When reared on *B. dorsalis*, *F. arisanus* produced 199 eggs per female and *D. tryoni* produced 50 eggs per female on *C. capitata*. When reared on its ancestral host (*B. tryoni*) in an artificial diet, *D. kraussii* produced 112 offspring; on *B. oleae* in olives, the result was much lower, at 23 offspring. *Anastrepha ludens* trials were conducted in mango fruits, *B. oleae* trials in olives and *B. tryoni* on artificial diet [19,22,24,31]. 

#### 6.3.2. Longevity

Adult longevity is an important attribute of parasitoid utility [20]. At 25–26 °C and 60% RH, *F. arisanus* had the greatest longevity (69 days) of the parasitoids potentially available for use in augmentative biological control in Australia. The next most long-lived species is *D. longicaudata* (51 days). The native species *D. kraussii* and *D. tryoni* have shorter lifespans at 30 and 26 days, respectively. Adult *F. arisanus* that emerged from *C. capitata* and *A. serpentina* had shorter life spans of 54 and 49 days, respectively, indicating host has an effect on longevity of the adult parasitoid [22,24,117]. Pre-release feeding can increase adult longevity and subsequent parasitoid success in the field [118]. A related biological attribute to longevity is generation time. *Diachasmimorpha longicaudata* and *D. tryoni* had similar generation times (23 and 22 days, respectively), whilst *F. arisanus* was almost double (42 days). The longer generation time of *F. arisanus* makes field establishment more difficult than for the other two species [24].

#### 6.3.3. Ease of Rearing

Whilst the egg parasitoid *F. arisanus* may give better fruit fly control in the field as it attacks earlier in the lifecycle of the pest than do the other larval parasitoid species [19], it is known to be difficult to culture [119]. *Diachasmimorpha tryoni* is known to be amenable to mass-rearing as large numbers (4.1 million/week) have been achieved in production facilities in Hawaii [50]. Similarly, both *D. kraussii* [40] and *D. longicaudata* [120] are suitable for mass-rearing. Furthermore, although mass releases of *D. tryoni* and *D. kraussii* have not been tested against *B. tryoni*, they have led to the successful control of other fruit flies including *C. capitata*. 

## 7. Conclusion

*Bactrocera tryoni* poses an enormous threat to the sustainability of Australian horticulture. In particular, the Fruit Fly Exclusion Zone (FFEZ) which provides *B. tryoni*-free areas permitting Australian producers to export to areas which are climatically favourable to *B. tryoni* and therefore, are susceptible to outbreaks. If *B. tryoni* cannot be managed effectively in both the FFEZ and RRZ, Australia’s economy may suffer from limited exports, as well as widespread crop damage. A solution to the over reliance on chemical insecticides is to develop a more integrated system to control populations in the RRZ and other endemic areas and stop their incursion into the FFEZ and other major endemic horticultural production areas. Parasitoids offer an attractive means of achieving this. 

Of the four parasitoid species currently available in Australia for possible use in an augmentative biological control program against *B. tryoni*, *D. longicaudata* and *F. arisanus* have the longest adult longevity and highest fecundity. However, as they cannot survive below 15 °C, they are probably unsuitable for Australia’s major horticultural production regions within the FFEZ and surrounding RRZ in inland NSW, Australia. These species also parasitise a wide range of fruit fly species and are therefore more likely to constitute a risk to non-target tephritid species, including natives and any introduced for biological control of weeds. The native parasitoids *D. kraussii * and *D. tryoni* were the only *B. tryoni* parasitoids detected in a survey undertaken in inland NSW [23] and are thus likely to be better suited climatically to this region. *Diachasmimorpha tryoni* appears to be better adapted to cooler climates than other parasitoids [50] (Table 4) and could therefore be released early in the season before large *B. tryoni* populations become established, or to deal with localised, early season outbreaks. Mature larvae of *D. tryoni* enter a winter diapause within host puparia [44] and emerge at the beginning of the season at the same time that *B. tryoni* emerge. Surveys have shown that *D. tryoni* is more abundant at higher elevations (greater than 600 metres) where temperatures are generally lower than at sea level [46]. The distribution of *D. tryoni* is similar to that of *B. tryoni* and occurs in all commercial fruit crops (except pineapple and strawberries) and many vegetable crops [121].

**Table 4:** Comparison of characteristics for the two Australian native parasitoids of *Bactrocera tryoni* (Adapted from [18,29,30,40,46]).

Characteristic	Australian native parasitoids of *Bactrocera tryoni*	
	*Diachasmimorpha tryoni*	*Diachasmimorpha kraussii*
Life stage attacked	Larva	Larva
Temporal pattern	Detected early in the season (cold tolerance)	Detected late in the season (heat tolerance)
Geographical pattern	Found in areas of higher elevation.	Relatively large geographical range
Adult longevity	15–25 days	15–30 days
Previous use on augmentative release	Previous success in Hawaii and Mexico	Previous success in Hawaii.

Importantly for augmentative releases, *D. tryoni* is known to be amenable to mass-rearing [50]. Additionally, although mass releases of *D. tryoni* have not been tested against *B. tryoni*, they have led to the successful control of other fruit flies such as *C. capitata*. Being suited to mass release in inland NSW is attractive because a widespread augmentative biological control program could decrease *B. tryoni* pest pressure on the FFEZ and also be used against isolated outbreaks within the FFEZ.

While the available information supports the viability of augmentative releases of *D. tryoni* against *B. tryoni* in inland NSW, there are large knowledge gaps concerning mass-rearing techniques, host specificity, and success rates. Although it is native to Australia, *D tryoni* has never been mass-reared for release in Australia or been field tested under Australian conditions. This information is critical to the success of biological control. Further, techniques that could optimise the mass-rearing process, such as an optimal food source for pre-release feeding and the influence of *B. tryoni* host size to maximise longevity and fecundity, have not been extensivelt explored for *D. tryoni* although literature is available for closely related species that indicates appropriate methods to fill these knowledge gaps. There have been non-target effects from the release of *D. tryoni *on gall forming fruit flies introduced to Hawaii as biological control agents of the weed *L. camara* [122]. Yet because *D. tryoni* is native to eastern Australia, where no gall forming insects have been recorded as hosts [110], they are unlikely to have equivalent non-target effects in Australia. *Lantana camara* is a pest weed in Australia, however, the biological control agents used to control it do not include gall-forming tephritids [123]. 

Like most parasitoids, *D. tryoni* is susceptible to insecticides even at exposure rates well under the recommended field application rates used for *B. tryoni* control [47]. Insecticides known to be harmful to *D. tryoni*, include carbaryl, permethrin and malathion [46]. The application of insecticides to control *B. tryoni* or other insect pests could significantly reduce the success of biological control against *B. tryoni* [47] and this needs to be considered in integrated pest management strategies. Targeted bait spraying is potentially less harmful to natural enemies because widespread application of toxins is avoided, however, baits containing sugars are potentially attractive to adult parasitoids. The combination of GF-120 bait sprays and biological control using *D. tryoni* has been described as a compatible control option [124]. The field effect of baits such as GF-120 has never been tested with *D. tryoni*, although mortality is known from direct exposure in laboratory conditions [124]. Thus, further research is required.

A more harmonious combination of control methods for *B. tryoni* is the release of parasitoids together with SIT [125]. These two techniques complement each other because they act on two different stages of *B. tryoni* (larvae and mating adult). A synergistic suppressive action can lead to local pest eradication [89]. This occurs as parasitoids tend to have a greater impact on relatively dense host populations because hosts are easy to locate and the parasitoid is able to reproduce efficiently. In contrast, SIT is expensive to use against large, dense pest populations but becomes more cost effective at lower pest densities [126]. Thus, parasitoids are generally released before sterile insects as parasitoids will suppress pest populations and reduce the number of sterile insects needed to achieve acceptable over-flooding ratios [46]. Success with SIT and biological control has been analysed in Mexico with *D. longicaudata* [56]. The use of both techniques helped to create fly-free zones, which allowed access to new markets valued at US $15 million [56]. There is evidence of this synergistic relationship in studies with *F. arisanus* and *D. kraussii* [40] and in the control of other insect pests such as codling moth [10]. Research into IPM strategies involving SIT and *D. tryoni *(or indeed other parasitoid species), however, have not been conducted in Australia and should be further researched to establish the ability of combining SIT and other biological control methods to eradicate and/or suppress *B. tryoni* in the FFEZ and RRZ.
